# Immunogenic cell death-related classification reveals prognosis and effectiveness of immunotherapy in breast cancer

**DOI:** 10.1038/s41598-024-52353-4

**Published:** 2024-01-23

**Authors:** Lei Zhu, Yanmei Wu, Haichun Zhao, Zicheng Guo, Biao Bo, Li Zheng

**Affiliations:** 1 Department of General Surgery, Panjin Liao-Oil Field Gem Flower Hospital, Panjin, 124000 China; 2 Department of Rheumatology and Immunology, Panjin Liao-Oil Field Gem Flower Hospital, Panjin, 124000 China

**Keywords:** Cancer, Cell biology, Computational biology and bioinformatics, Biomarkers, Diseases, Oncology

## Abstract

Lack of specific biomarkers and effective drug targets constrains therapeutic research in breast cancer (BC). In this regard, therapeutic modulation of damage-associated molecular patterns (DAMPs)-induced immunogenic cell death (ICD) may help improve the effect of immunotherapy in individuals with BC. The aim of this investigation was to develop biomarkers for ICD and to construct ICD-related risk estimation models to predict prognosis and immunotherapy outcomes of BC. RNA-seq transcriptome information and medical data from individuals with BC (n = 943) were obtained from TCGA. Expression data from a separate BC cohort (GEO: GSE20685) were used for validation. We identified subtypes of high and low ICD gene expression by consensus clustering and assessed the connection between ICD subtypes and tumor microenvironment (TME). In addition, different algorithms were used to construct ICD-based prognostic models of BC. BC samples were categorized into subtypes of high and low ICD expression depending on the expression of genes correlated with ICD. The subtype of ICD high-expression subtypes are correlated with poor prognosis in breast cancer, while ICD low-expression subtypes may predict better clinical outcomes. We also created and verified a predictive signature model depending on four ICD-related genes (*ATG5, CD8A, CD8B*, and *HSP90AA1*), which correlates with TME status and predicts clinical outcomes of BC patients. We highlight the connection of ICD subtypes with the dynamic evolution of TME in BC and present a novel ICD-based prognostic model of BC. In clinical practice, distinction of ICD subtype and assessment of ICD-related biomarkers should help guide treatment planning and improve the effectiveness of tumor immunotherapy.

## Introduction

Immunogenic cell death (ICD) is the mechanism by which, following a death stimulus, tumor cells transition from a non-immunogenic to an immunogenic state to induce an anti-tumor immune response^1,2^. ICD is characterized by the release of a cascade of signaling molecules referred to as damage-associated molecular patterns (DAMPs). These include calreticulin, which is found on the surface of cells, high mobility group proteins (HMGB1), which are secreted by cancerous cells, and ATP molecules and heat shock proteins (HSP70, HSP90), which are produced by cells undergoing death. DAMPs released during ICD can trigger a series of cytological responses by binding to pathogen recognition receptors (PRRs) in the dendritic cells’ surface, ultimately activating the innate and adaptive immune reactions^3–5^. The identification of ICD as a crucial mechanism mediating tumor-directed immune responses provided a new direction for precision tumor treatment^6,7^. Moreover, conclusive evidence emphasizes that such mechanism supports long-term efficacy of anticancer drugs, providing therefore novel prospects for immunogenic therapies^2,8^. Interestingly, although many ICD-based clinical models have been studied, the therapeutic value of ICD in medical practice is not yet fully considerable^5^. Therefore, there is a clear need to further investigate the mechanisms underlying ICD and to discover ICD-related biomarkers predictive of immunotherapy response in cancer patients.

Breast cancer (BC) is the primary reason for mortality connected to cancer among women worldwide^9,10^. Despite continuous medical advances, clinical outcomes for breast cancer patients still need improvement^10,11^. Research suggests that escape from immune surveillance by blockade of immune checkpoints is a fundamental mechanism by which tumor cells prevent T cell-mediated antitumor responses^12–14^. Hence, diverse immunotherapeutic strategies, including immune checkpoint inhibitors and tumor vaccines, are eagerly explored to better treat patients with BC^15–19^. In this regard, the identification of ICD-related markers at both pre- and post-treatment stages may prove invaluable to help guide treatment choices and greatly aid the development of patient-tailored immunotherapies.

In this work, we detected ICD-based markers of BC and constructed a risk signature depending on ICD to assess tumor microenvironment (TME) characteristics, prognosis, and immunotherapeutic profiles of BC patients. Our findings provide novel insights to help advance the clinical application of ICD-based tumor immunotherapy.

## Materials and methods

### Datasets and statistical analysis

RNA-seq transcriptome information and corresponding clinicopathological data of 943 breast cancer patients were obtained from TCGA (https://portal.gdc.cancer.gov/) and used as a training set. The Gene Expression Omnibus (GEO) GSE 20,685 dataset (https://www.ncbi.nlm.nih.gov/geo/query/acc.cgi?acc=GSE20685)^20,21^ provided gene expression and informative clinical data on 327 breast cancer patients and used them as validation set.

Log2 normalization was used to transform all gene expression data. A two-group t-test was employed to compare normal and tumor tissues, with a P-value < 0.05 suggesting statistical significance. The Spearman or Pearson assessment was used for scrutinizing the closeness between two variables; P < 0.05 was regarded statistically significant. R program was employed to perform all statistical analyses (version 4.2.0).

### Consensus clustering

We performed consensus clustering with the “ConsensusClusterPlus” instrument in R program to identify the subtypes of molecules related to ICD. Additionally, we examined the model number of clusters between k = 2–10 and conducted the procedure 1000 times to confirm the accuracy of the results. The “pheatmap” function in R was employed to generate a cluster map.

### Analysis of differentially expressed genes (DEGs)

Differential expression of mRNAs with screening criteria: | fold change|> 2 and adjusted P < 0.05 was evaluated utilizing the “limma” package in R (version 4.2.0). The adjusted P values were assessed to reduce errors in TCGA data.

### Functional enrichment analysis

Gene Ontology (GO) and Kyoto encyclopedia of genes and genomes (KEGG)^22,23^ analyses were performed to obtain and compare differentially enriched signaling mechanisms and biological functions between subtypes of low and high ICD. The ''clusterProfiler'' package in R program was utilized to assess GO and KEGG pathways^24^. Criteria for GO and KEGG enrichment analysis included the consideration of Q-value and P-value levels below 0.05.

### Gene set enrichment analysis (GSEA)

GSEA software was utilized to analyze enrichment properties of the MSigDB set and to define whether there were significant variations among gene expression sets between high and low ICD subtypes.

### Characterization of tumor immune microenvironments

To characterize the immune microenvironment in BC samples, the CiberSort algorithm (https://cibersort.stanford.edu/) with 1000 iterations was employed to examine expression data from samples of cancers^25^. Relative proportions of immune cell kinds in the subgroups of high and low ICD were thus obtained and visualized as a landscape map.

### Somatic mutation analysis

The database of TCGA was utilized to obtain the BC samples’ somatic mutation data and the "MafTools" in R was employed to construct waterfall plots to facilitate visualization and summarization of mutation information.

### Survival analysis

The "survminer" and "survivor " packages in R were employed with Kaplan–Meier (KM) analysis to compare the overall survival (OS) between subgroups of low and high ICD risk. Prognostic indicators were determined by univariate COX analysis, and multivariate COX analysis was utilized to define whether the risk score was an independent OS risk factor.

### Construction of the ICD-related risk signature

To define the coefficient values for every identified correlation, genes correlated with immunity considered statistically significant in univariate COX regression analysis were selected for analysis of Lasso Cox regression. Lasso regression is well known for its ability to improve the prognostic ability and interpretability of statistical models by combining variable selection and regularization.

### Immunotherapy response

Immunotherapy response was assessed by Tumor Immune Dysfunction and Exclusion (TIDE) analysis. The analytical technique, TIDE (http://tide.dfci.harvard.edu/), is utilized for anticipating immunotherapy response assessing cancer immune evasion pathways, like T cell malfunction and suppression of T cell infiltration in malignancies with reduced CTL levels.

### Drug sensitivity analysis

Drug sensitivity data and gene expression profiles were evaluated by accessing the database of CellMiner (https://discover.nci.nih.gov/cellminer/home.do). The R packages “imput” and “limma” were employed for drug sensitivity assessment, whereas the “ggplot2” and “ggpubr” packages were utilized to illustrate the correlation between drug sensitivity and gene expression level.

### Cell culture

Human normal breast (MDA-KB2) and BC cells (MDA-MB-231, MCF-7, and BT549) were acquired from Procell Life Science & Technology Co, Ltd (Wuhan, China). The cell lines were cultivated in DMEM treated with 1% penicillin, 10% FBS, and 1% streptomycin. The cells were incubated with 5% CO_2_ at 37 ℃.

### Quantitative real-time PCR (qRT-PCR)

TRIzol reagent (Life Technologies, USA) was employed to obtain total RNA from cells following the manufacturer's directions.NanoDrop 2000 was used to determine the concentration of RNA. Subsequently, PrimeScript RT Master Mix (Takara, Japan) was employed to synthesise cDNA. cDNA was synthesised utilizing TB Green Premix Ex Taq (Takara, Japan) on a System of ABI PRISM 7900 Sequence Detection (Applied Biosystems, Carlsbad, USA) to complete qRT-PCR (Table 1).

**Table 1:** qPCR primer sequences.

Gene	Forward primer	Reverse primer
HSP90AA1	CACAGGTGAGACCAAGGACC	TTCCCCTAGTTTTCATGCCACA
β-actin	CATGTACGTTGCTATCCAGGC	CTCCTTAATGTCACGCACGAT

### siRNA sequence

The siRNA targeting HSP90AA1 and the negative control si-NC were designed and synthesized by GenePharma (Shanghai, China), the transfection complexes formed by siRNA and transfection reagents were configured according to the manufacturer's instructions. 100 μl of the transfection mixtures were added to the different groups, and after shaking, the cells were static cultured at 37 °C and samples were collected and detected for mRNA to verify the target knockdown efficiency at 24–72 h. The siRNA sequences were as follows: siRNA1F: 5′-UUUUGUUGAGCUCUUCUUGAU-3′, R: 5′-CAAGAAGAGCUCAACAAAACA-3′; siRNA2F: 5′-AUUACUAGCUCUGCUUUAGUG-3′, R: 5′-CUAAAGCAGAGCUAGUAAUGC-3′.

### Western blot analysis

RIPA buffer comprising a phosphatase inhibitor cocktail was utilized to lysis the cells After completion of cell transfection. Proteins were subjected to a transfer process to nitrocellulose membranes after being loaded and electrophoretically separated on SDS polyacrylamide gel electrophoresis (SDS-PAGE). The primary antibodies were introduced to facilitate the binding process with the respective proteins, and incubated overnight at 4 °C. Following this, the membrane was subjected to one hour of incubation with a secondary antibody conjugated with HRP obtained from Absin, a company based in Shanghai, China at room temperature. Subsequently, the membrane was exposed to an ECL reagent obtained from NCM Biotech, a company located in Suzhou, China.

### CCK8 assay

MDA-MB-231, MCF-7 and gene knockdown BC cells were inoculated into 96-well plates in equal numbers. At a ratio of CCK-8 solution: medium = 10:100, 10 µL of CCK solution was introduced to the cells. Following a 2-h incubation period, a multifunctional microplate reader was employed with a wavelength of 450 nm to assess the values of optical density (OD).

### Wound healing assay

The cells’ migratory efficiency was observed utilizing the assays of wound healing, and knockdown breast cancer cells reached 100% confluence with a 10 µl pipette to produce a wound in the centre of the cell monomer. Subsequently, the culture was continued in the incubator, and the Image J program was utilized to calculate the wound area at the same intervals.

### Cell invasion assay

Cell invasion efficiency was assessed using the The cells that underwent transfection were gathered and suspended in a medium without serum. Subsequently, about 2 × 10^4^ cells were introduced into the top chamber along with 300 µL of serum-free DMEM. In contrast, the bottom chamber was supplied with 500 µL of DMEM containing 10% FBS. Following a period of 48 h, the cells that attached to the bottom membrane were then treated with a 4% paraformaldehyde solution for fixation purposes. Following fixation, the cells were subjected to staining using crystal violet. Residual cells were eliminated from the surface of the top membrane with the use of a cotton swab. Subsequently, the cells were captured in photographs and subjected to analysis with a light microscope.

### Statistical analysis

The two-tailed unpaired analyses were employed to conduct the statistical analyses. GraphPad Prism program was employed for statistical analysis. P < 0.05 was regarded statistically significant. R program was utilized to conduct all data analysis (version 4.1.0).

## Results

### Cluster analysis to identify ICD-related subtypes in breast cancer

**Figure 1:**
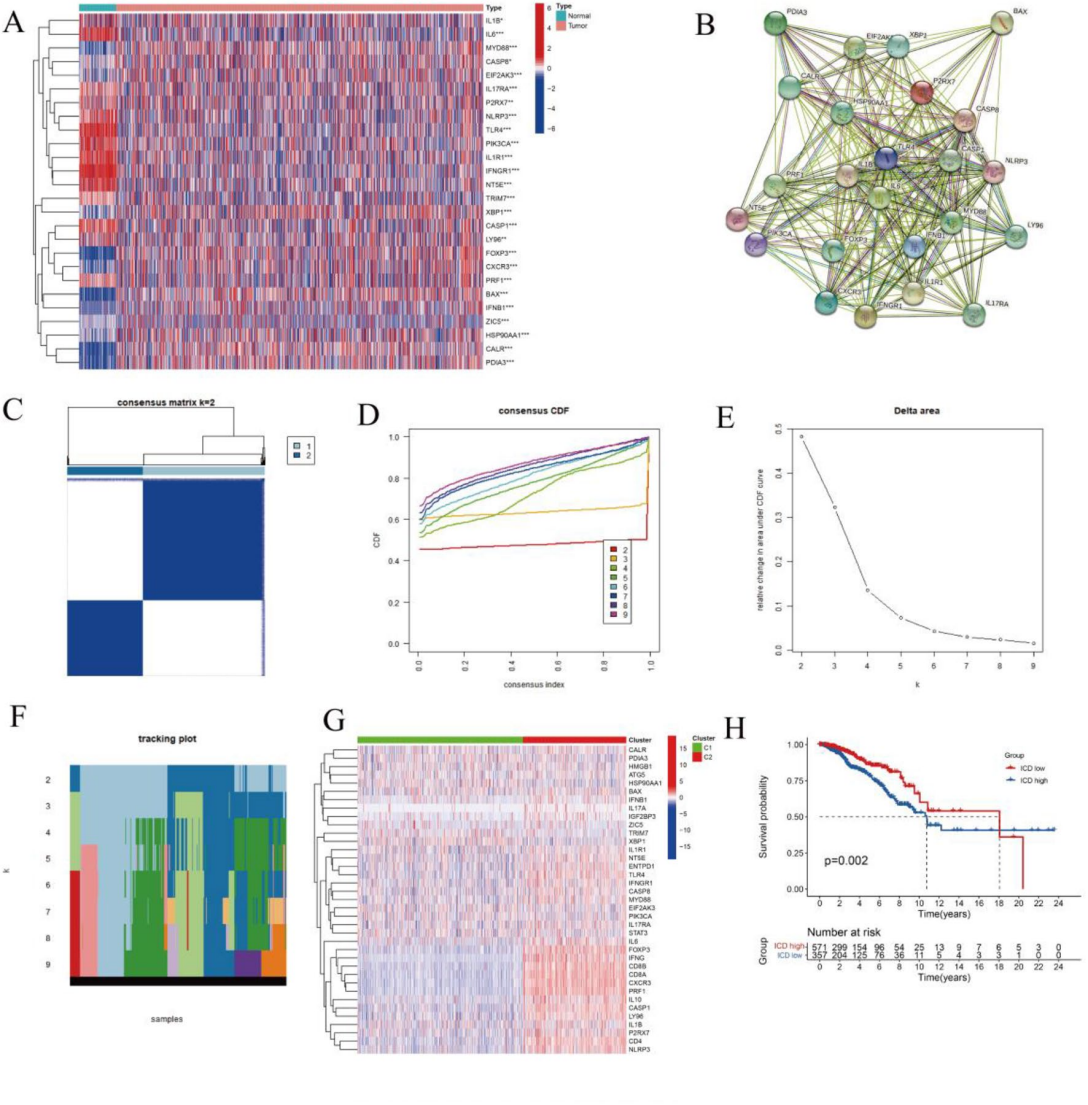
Detection of ICD-related subtypes in BC. (**A**) Differential expression profiles of ICD-related genes in normal breast and BC samples from TCGA database. (**B**) Protein–protein interaction network of ICD-related genes. (**C–F**) Consensus clustering scheme for genes related to ICD in BC samples (k = 2). (**G**) Heat map depicting differential expression of 41 genes related to ICD and their different isoforms. Red denotes high expression and blue represents low expression. (**H**) Kaplan–Meier curves of OS in high and low ICD expression subtypes.

We analyzed the ICD genes expression in 943 healthy and BC tissues obtained from TCGA (Fig. 1A). Most ICD genes, including PDIA3, CALR, CXCR3, FOXP3, MYD88, and XBP1, among others, were highly expressed in BC. However, several ICD genes, including TLR4, PIK3CA, IFNGR1, IL1R1, were also highly expressed in normal tissues. In addition, the linkage between these genes correlated with ICD was demonstrated utilizing protein–protein interaction network analysis, which was performed on the database of STRING (Fig. 1B).

Therefore, the technique of consensus clustering was used in order to discover clusters related to BC that are connected with ICDs. K-means clustering results suggested that the two clusters in the TCGA cohort have various patterns of ICD gene expression (Fig. 1C–F). The cluster of C1 represents the ICD-low subtype, as it exhibits low ICD gene expression levels (Fig. 1G). Correspondingly, the C2 group was defined as the subtype of high ICD based on higher ICD gene expression levels (Fig. 1G). Additionally, survival analysis exhibited that these subtypes of ICD demonstrated various medical outcomes. Generally, the subtype with high ICD had a poor prognosis. In contrast, the subtype of low ICD was connected to promising medical results (Fig. 1H), which may be attributed to the activation of ICD-related genes for immune signaling.

### Differentially expressed genes (DEG) and enrichment analysis based on ICD subtypes

**Figure 2:**
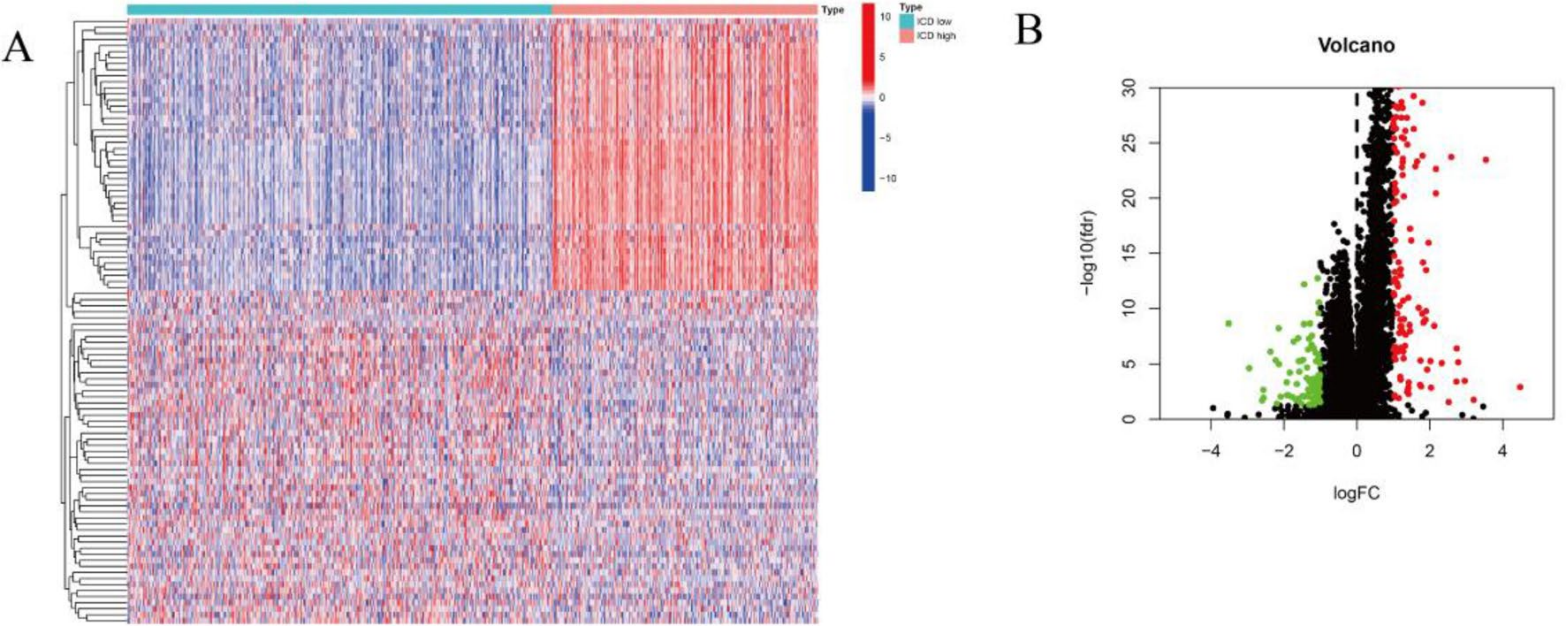
Analysis of differential gene expression in subtypes of high and low ICD expression. (**A**) Heat map showing differential expression of ICD-related genes, with red representing high expression and blue demonstrating low expression. (**B**) Volcano plot showing DEG distribution between ICD-high and ICD-low subtypes in the TCGA cohort.

**Figure 3:**
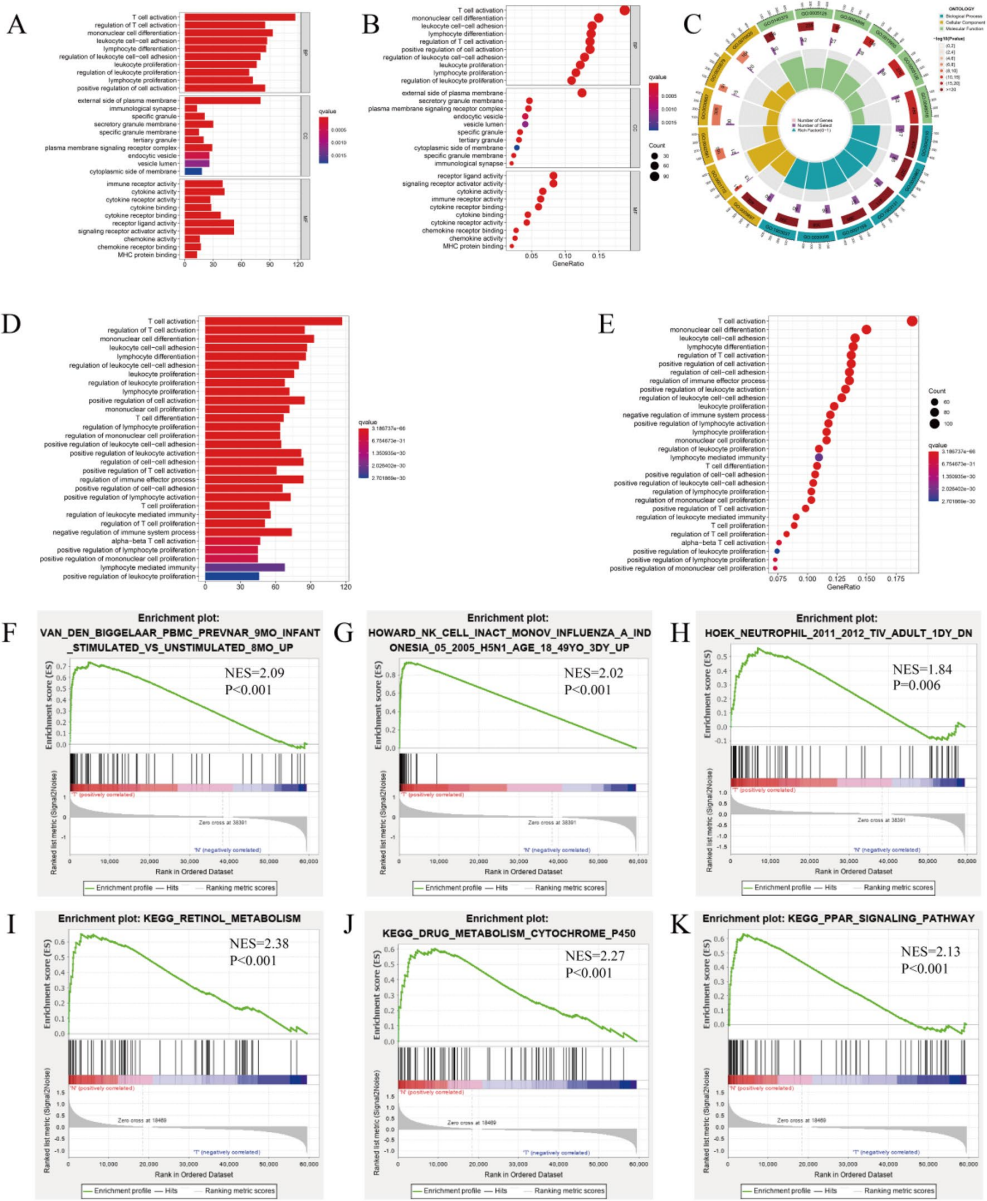
Functional enrichment analysis of differentially expressed ICD-related genes. (**A–C**) GO enrichment analysis. (**D,E**) KEGG pathways analysis. Dot sizes represent gene numbers, and dot color represents − log10 (adjusted p-value). (**F–K**) GSEA results depicting signaling pathways associated with the ICD-high and -low subtypes.

To discover the molecular prognostic differences determinants between ICD-low and -high subtypes, we conducted DEG analysis and identified the major signaling pathways enriched in each subtype. Typically, 167 DEGs were identified (Fig. 2A, B). Corresponding GO and KEGG analyses revealed that genes upregulated in the subtype with high ICD are enriched in activities connected with immunity, such as cytokine and cytokine receptors, T cell activation, lymphocyte proliferation, MHC protein binding, chemokine receptor binding, and adhesion correlated with the leukocyte cell (Fig. 3A–E). These outcomes suggest that up-regulation of relevant ICD genes in high ICD BC subtypes may affect the immune response and have an impact on the prognosis of tumor patients by promoting a more suppressive tumor microenvironment.

We further predicted the signaling mechanisms triggered in ICD-high tumor subtypes using GSEA. The corresponding gene sets were correlated with several immune mechanisms, like cytotoxicity mediated by the natural killer (NK) cell and neutrophil-mediated inflammatory response (Fig. 3F–H), as well as with other pathways, such as retinol metabolism and drug metabolism (Fig. 3I–K).

### Somatic mutations analysis

**Figure 4:**
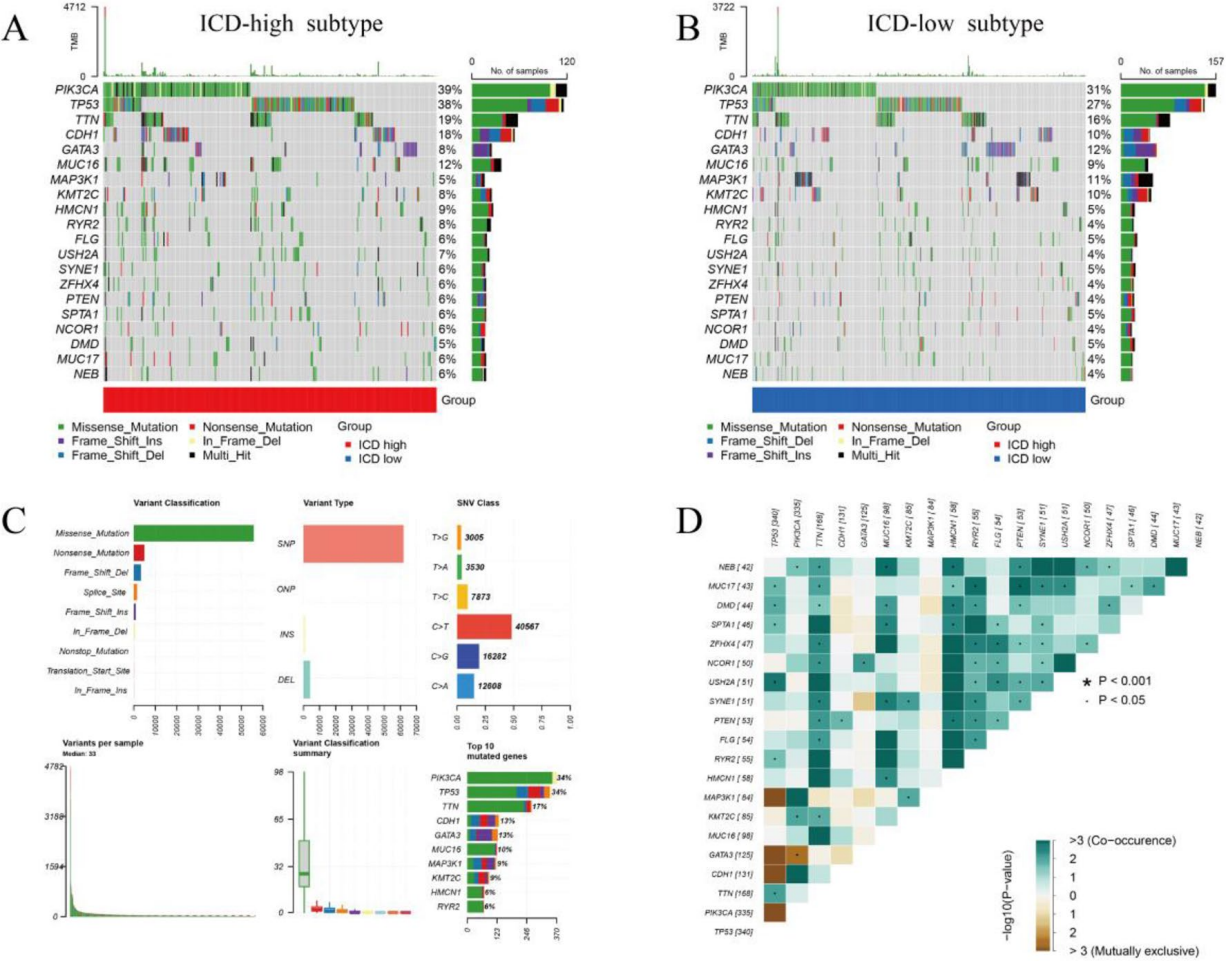
Comparison of somatic mutation profiles between the two ICD subtypes (**A,B**). Visualization of the top 20 mutated genes in high (**A**) and low (**B**) ICD subtypes of BC. (**C**) Analysis of somatic mutation types in the TCGA cohort. (**D**) Correlation analysis for the top 20 mutated genes. Brown represents negative correlation; green represents positive correlation; *P < 0.001, ^**.**^P < 0.05.

We examined somatic mutation characteristics in the different ICD subtypes. Although mutations in PIK3CA, TP53, TTN, and GDH1 were the most common alterations in both groups, mutation frequencies differed significantly. Specifically, the frequency of PIK3CA and TP53 mutations was higher in the ICD-high subtype, accounting for 39% and 38% of the total mutations, respectively, while the corresponding frequencies in the ICD-low subtypes were 31% and 27% (Fig. 4A, B). Further, we noted that missense mutations are the most common alterations in the above genes, and that single nucleotide polymorphisms (SNPs) and C-T changes play a non-negligible role in these mutations (Fig. 4C). Results of correlation analysis of the most significant, mutated ICD genes are depicted in Fig. 4D.

### Immune microenvironmental landscape of ICD subtypes

**Figure 5:**
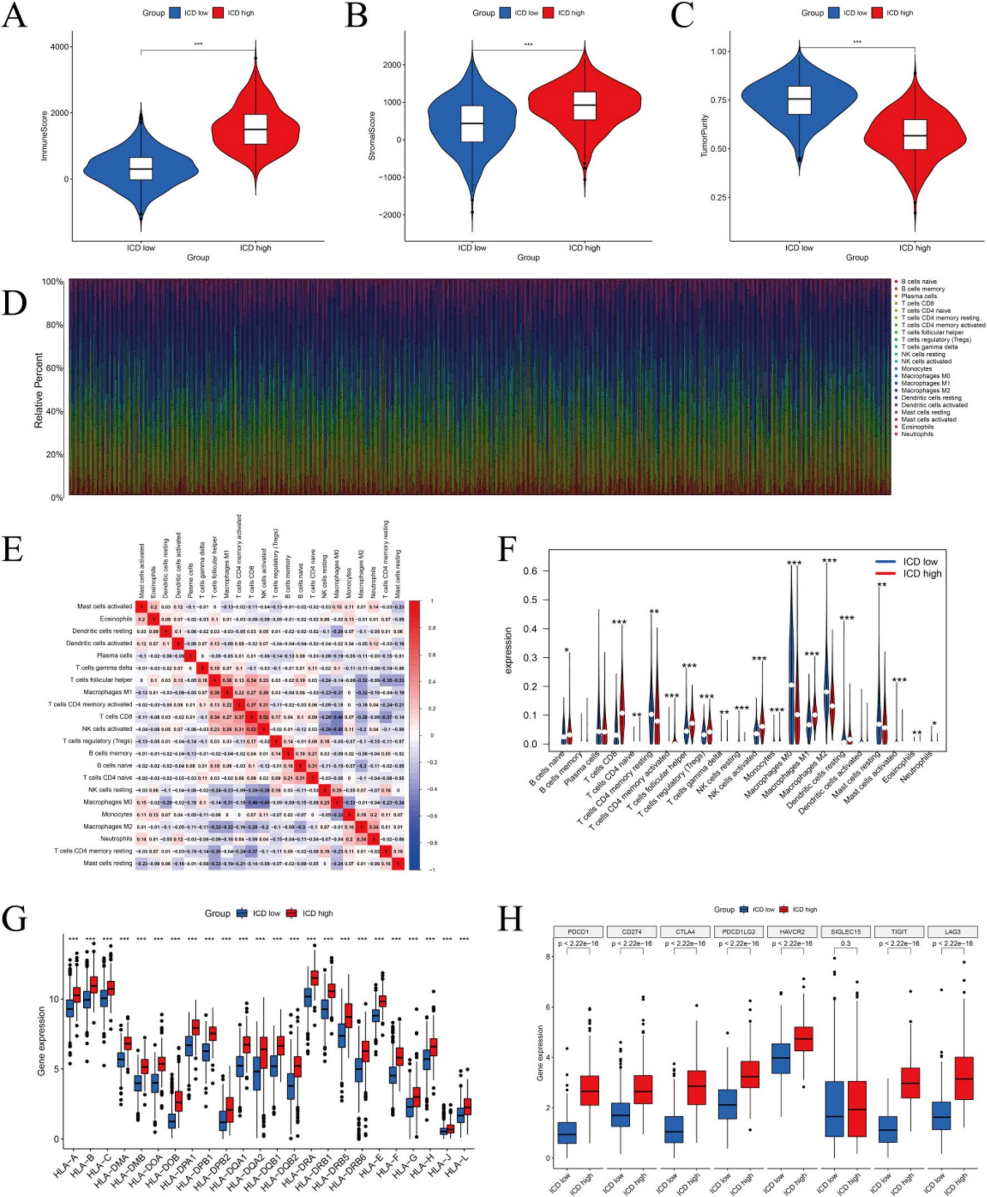
Immunological characteristics of the two ICD subtypes. (**A–C**) Violin plots presenting median and quartiles of immune score, stromal score, and tumor purity. (**D**) Comparison of the proportion of infiltrating immune cell types in the high and low ICD subtypes. (**E**) Correlation analysis between immune cells. Red denotes positive correlation and blue represents negative correlation. (**F**) Violin diagrams suggest significant variations in immune cell representation between high and low ICD subtypes. (**G,H**) Box plots show differential expression of multiple immune checkpoint (**H**) and HLA genes (**G**) between high and low ICD subtypes. ***P < 0.001, **P < 0.01, *P < 0.05.

There is compelling evidence that ICD can activate a number of antitumor immune responses. Our analysis revealed variations in the TME composition between high and low ICD subtypes in BC. The group with high expression of the ICD had elevated immune and stromal scores, while demonstrating decreased tumor purity. In contrast, the inverse was seen in the group with low expression of ICD. (Fig. 5A–C). Subsequently, we conducted an assessment of variations in the infiltration levels of 22 distinct immune cell types within the ICD subtypes. This analysis was carried out employing the CiberSort method and LM22 characteristic matrices. The results revealed distinct profiles among BC samples (Fig. 5D). Patients with a tumor subtype of high ICD had a significantly elevated proportion of NK cells, follicular helper T cells, CD8+ T cells, and activated and M1 macrophages (Fig. 5F). Additionally, we evaluated the correlation between immune cell types and detected a positive correlation between CD8^+^ T and NK cells (R = 0.52, P < 0.05) and a negative correlation between CD8^+^ T cells and M0 macrophages (R = 0.46, P < 0.05) (Fig. 5E). Notably, the low-ICD expression subtype had a significant downregulation of human leukocyte antigen (HLA) and immunological checkpoint genes. Conversely, the high-ICD group of tumors shown a reversal of this trend (Fig. 5G, H).

### Construction and verification of ICD risk signatures

**Figure 6:**
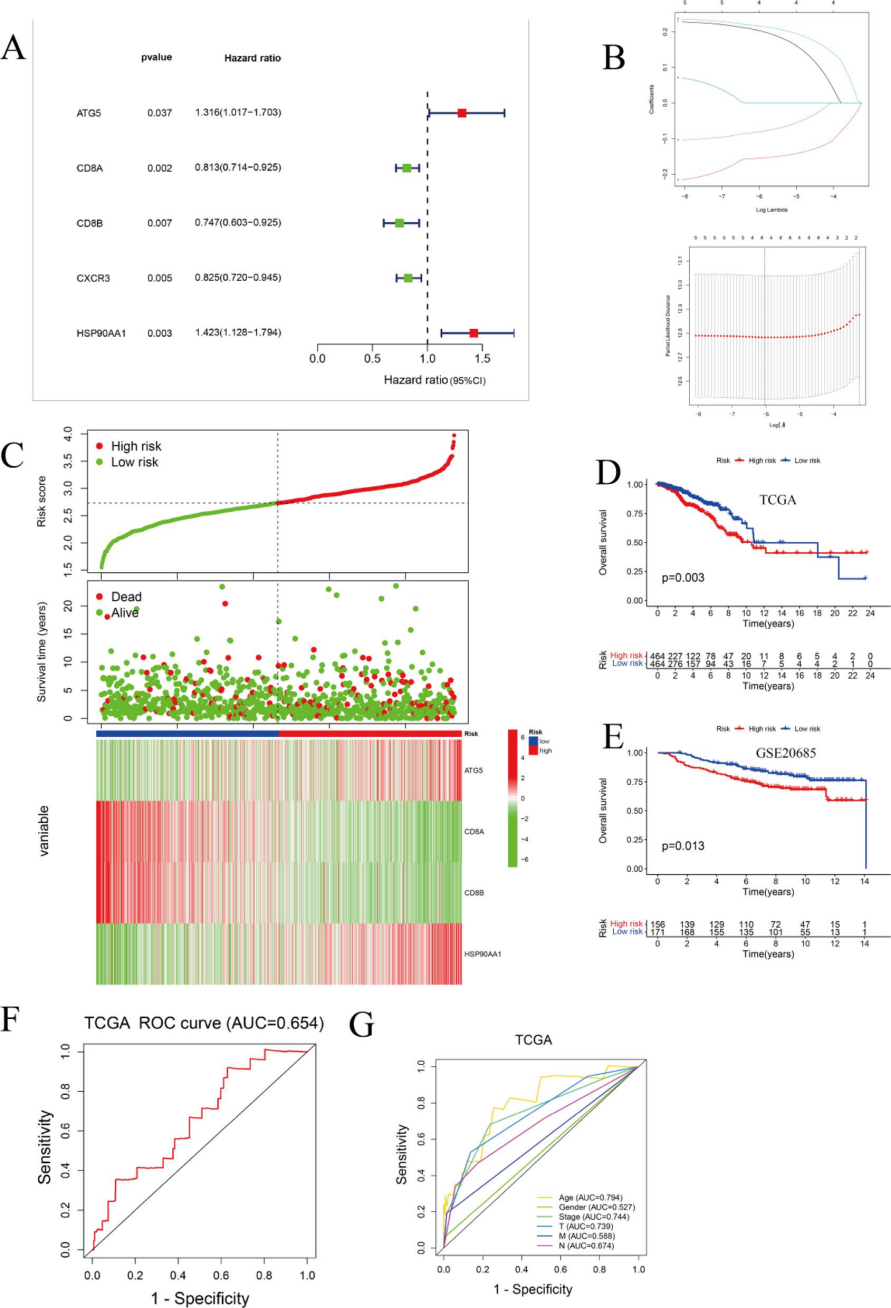
Construction and validation of the ICD risk signature. (**A**) Assessment of the predictive value of ICD genes for OS according to univariate COX analysis. (**B**) Identification of the four genes most connected with BC prognosis in the TCGA data cohort (Lasso Cox analysis). (**C**) Heat map of risk score distribution, patient survival status, and expression of the four most prognostically relevant ICD genes in the TCGA cohort. (**D,E**) Kaplan–Meier analysis demonstrates the predictive significance of the risk model in the TCGA and GSE20685 cohorts. (**F,G**) AUC curves reveal the accuracy of risk models in the TCGA cohort.

We built a predictive model that depended on genes correlated with ICD. Five genes correlated with ICD were found to be significantly connected to the OS of the patient in univariate COX analysis (Fig. 6A). Next, Lasso regression analysis was utilized to examine and select ICD-related genes for evaluation of predictive models (Fig. 6B). A risk signature was finally constructed with the following algorithm: risk-score = (0.2043)*ATG5 + (−0.1551)*CD8A + (−0.0813)*CD8B + (0.2170)*HSP90AA1. We next examined the relationship between risk score and status of survival. The outcomes revealed that the number of survival states was much lower in the group of high-risk in contrast to the group of low-risk (Fig. 6C). KM analysis was subsequently conducted to investigate the importance of this risk profile in BC (Fig. 6D, E). The outcomes exhibited that a high risk score in the TCGA cohort predicted poorer OS (Fig. 6D). Of note, this finding was confirmed in a separate GEO cohort (Fig. 6E). Moreover, AUC curves, created to verify the prognostic model accuracy (Fig. 6F, G), indicated good performance of the model in the cohort of TCGA (AUC > 0.6).

### Correlation analysis of ICD risk signature and TME

**Figure 7:**
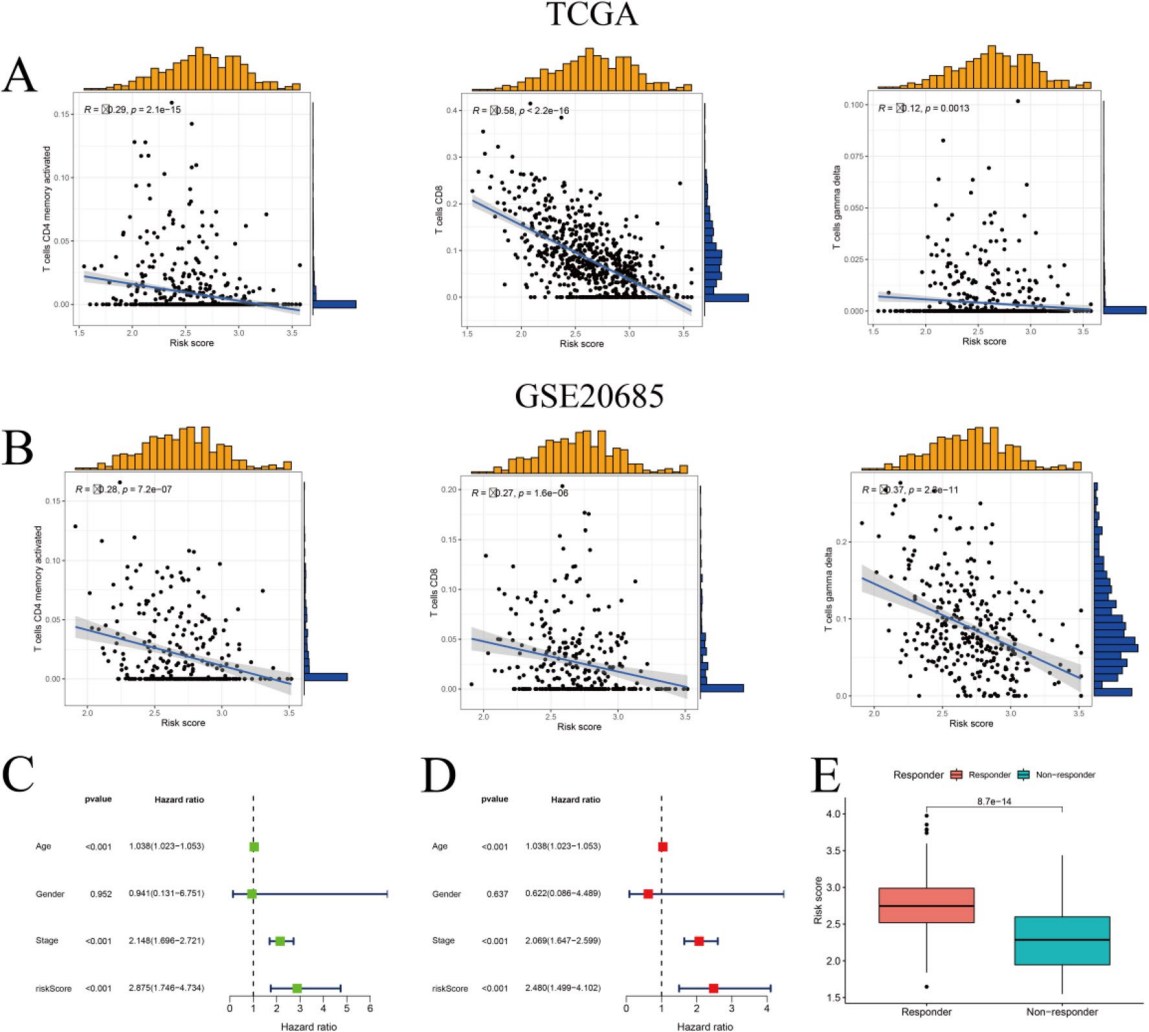
Relationship between ICD risk signature and TME. (**A,B**) Scatter plots show that risk scores correlate with infiltration of CD8^+^ T cells and activated CD4^+^ memory cells in both the TCGA (**A**) and the GSE20685 cohort (**B**). (**C,D**) Univariate and multivariate Cox analyses evaluating the independent prognostic value of the ICD risk signature in BC patients. (**E**) Box plot depicting the association of the ICD risk score with immunotherapy response.

In light of the significant influence of ICD on immune responses against tumors, our subsequent analysis aimed to evaluate the possible connection between our ICD risk score and the TME employing the CiberSort method. The findings of the study indicated a negative correlation between high-risk scores of tumors and the infiltration of CD8+ T and activated CD4+ memory T cells (Fig. 7A), the conclusion verified by a study of the GEO cohort. (Fig. 7B). Following that, we conducted an assessment of the independent predictive significance of the ICD risk score employing univariate and multivariate COX analyses. The results of the univariate COX analysis indicated a statistically significant correlation between a high ICD risk score and a poorer OS (Fig. 7C). Subsequently, the multivariate COX analysis demonstrated that the ICD risk score has the potential to function as an independent predictive variable for individuals with BC (Fig. 7D). Furthermore, the TIDE technique was employed to assess the possible efficacy of our ICD risk signature in relation to immune therapy response. In general, compared to the immunotherapy non-responder group, the group of subjects who responded to immunotherapy (low ICD risk) had higher scores and may predict that individuals with higher scores could derive more therapeutic benefit from immunotherapy (Fig. 7E).

### Drug sensitivity analysis

**Figure 8:**
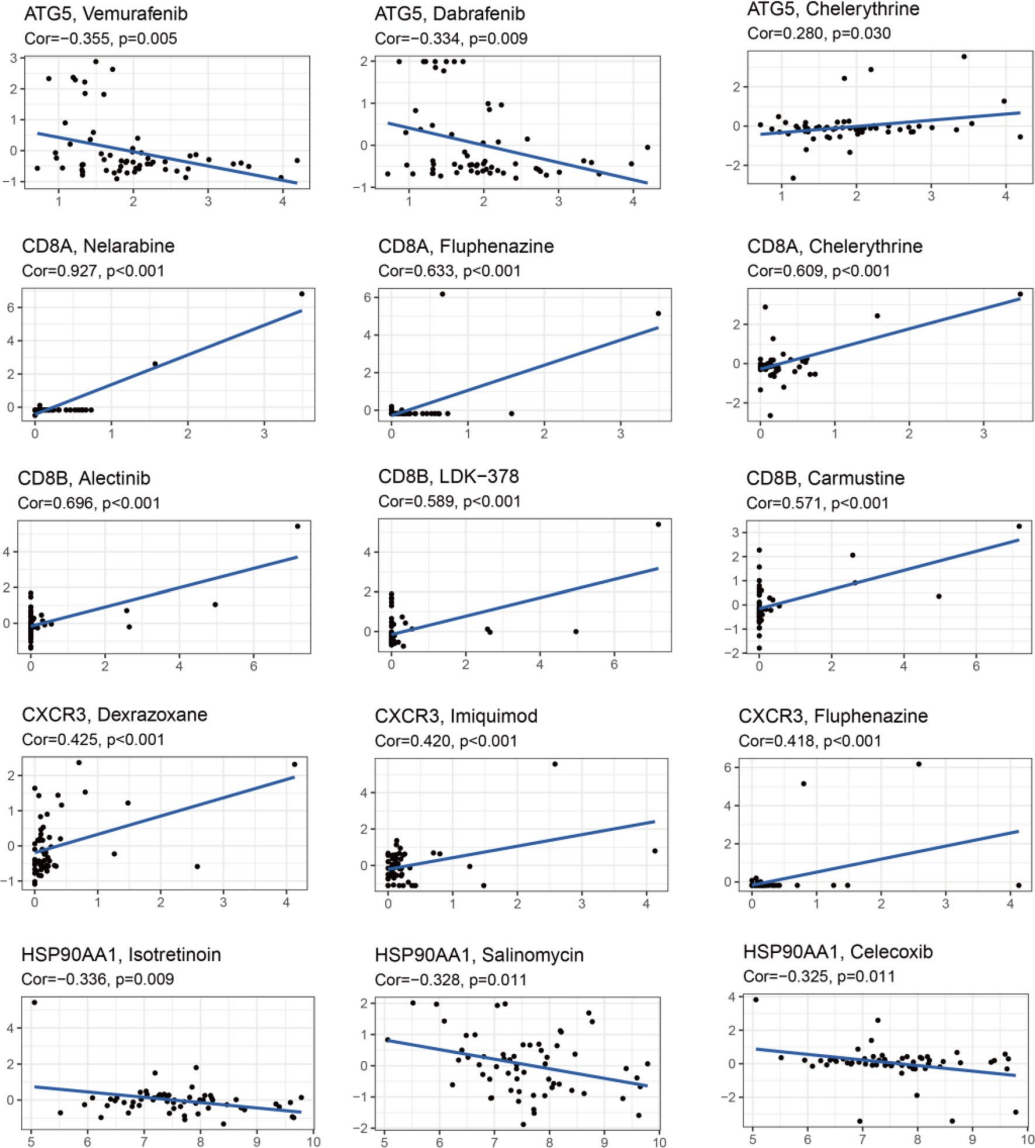
Drug sensitivity analysis of ICD genes associated with prognosis. Horizontal coordinates represent gene expression and vertical coordinates represent drug sensitivity scores; increased gene expression of ATG5 and HSP90AA1 resulted in decreased drug sensitivity.

Finally, we analyzed the drug sensitivity of BC prognosis-related genes using the CellMiner database (Fig. 8). Results suggested that vemurafenib, nelarabine, alectinib, dexrazoxane, and isotretinoin were the best matches for each of the five prognosis-related genes. Noteworthy, horizontal coordinates represent gene expression and vertical coordinates represent drug sensitivity scores; increased gene expression of ATG5 and HSP90AA1 resulted in decreased drug sensitivity.

### Knockdown of HSP90AA1 inhibits breast cancer proliferation and migration

**Figure 9:**
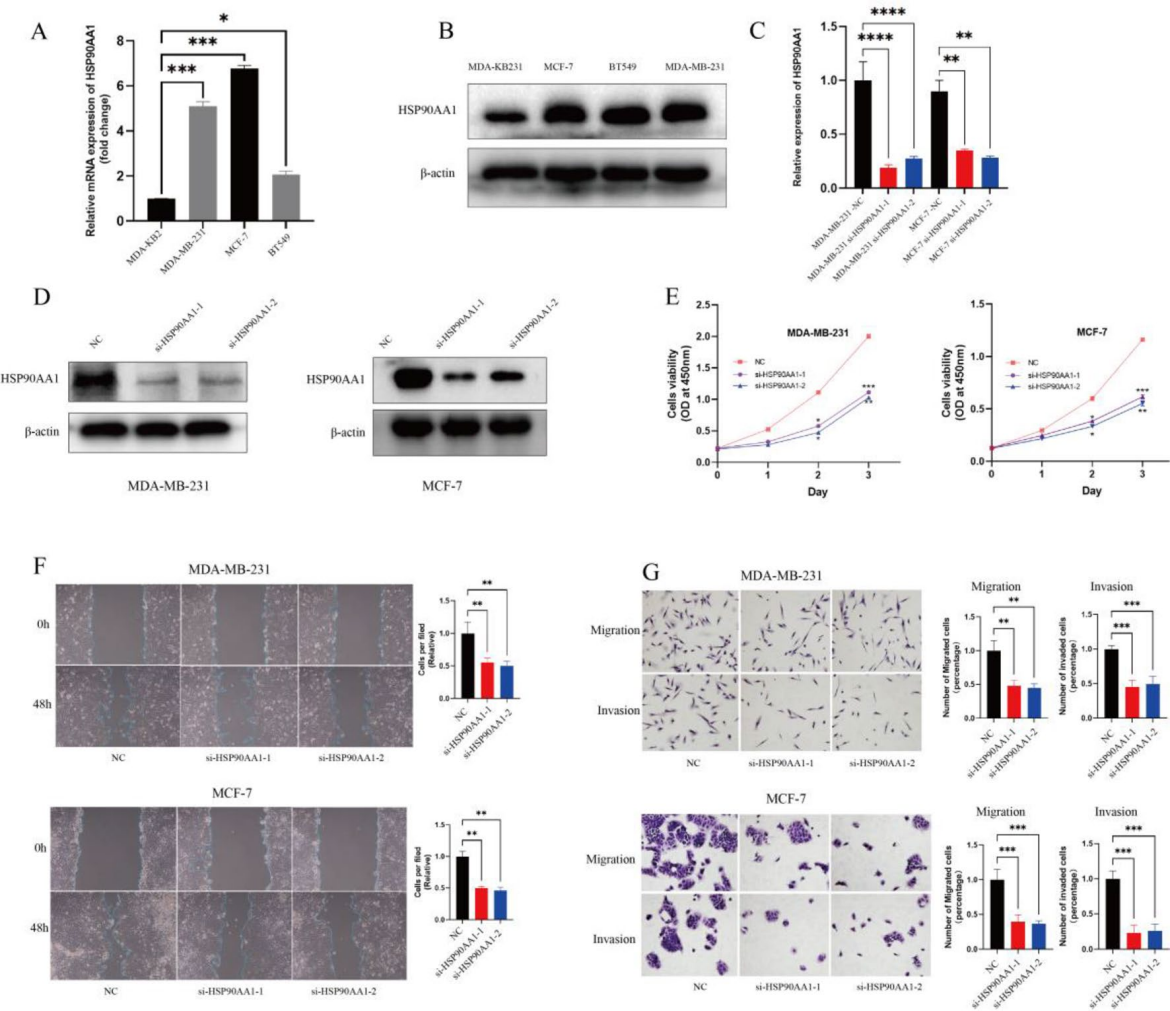
Knockdown of HSP90AA1 inhibits breast cancer proliferation and migration. (**A,B**) Expression levels of HSP90AA1 in different breast cancer cells observed by qRT-PCR and Western blot. (**C,D**) RT-PCR and Western blot validate knockdown efficiency of HSP90AA1. (**E**) CCKB assay confirms the effect of HSP90AA1 on the proliferative viability of breast cancer cells. (**F**) Effect of HSP90AA1 on the migration ability of breast cancer cells confirmed by wound healing assay. (**G**) Transwell assay confirms the effect of HSP90AA1 on the migration and invasive ability of breast cancer cells.

To explore the effect of breast cancer prognosis-related genes on tumor cell progression in the model, we selected the gene with the highest risk factor (HSP90AA1) for validation^26^. We observed the HSP90AA1 expression level in several breast cancer cells utilizing qRT-PCR and Western blot (Fig. 9A, B) (Supplementary Fig) and selected MDA-KB231 and MCF-7 cell lines for subsequent experimental validation. After completion of cell transfection, the knockdown efficiency of HSP90AA1 was validated employing qRT-PCR and Western Blot (Fig. 9C, D) (Supplementary Fig). Subsequently, CCK8 assay was utilized to explore the HSP90AA1 impact on the proliferative viability of BC cells, and the growth efficiency of cancer cells was significantly inhibited after HSP90AA1 knockdown (Fig. 9E). In the meanwhile, it was shown by the wound healing assay that the downregulation of HSP90AA1 also suppressed the migratory capacity of BC cells (Fig. 9F). Furthermore, the Transwell test was utilized to evaluate the migratory and invasive behavior of cancerous cells. When HSP90AA1 was knocked down, the BC cells’ migration and invasion were significantly reduced (Fig. 9G). Therefore, we believe that the HSP90AA1 overexpression favours the proliferation migration and invasion of BC cells.

## Discussion

ICD is regarded as a unique modulated type of cellular death owing to its capacity to initiate antigen-specific adaptive immune responses by means of the release of danger signals (i.e., DAMPs)^27–30^. Whereas traditional radiotherapy and chemotherapy are often insufficient for effective tumor control, the combination of immunogenic therapy and immune checkpoint inhibitors has shown promising results^2,31,32^. Consequently, the detection of markers correlated with ICD should help differentiate BC patients that may benefit from immunotherapy. The present work may involve in the development of targeted immune therapies for BC by describing a significant connection between the ICD-related gene expression and both the status of the TME and BC prognosis. Through the examination of differential gene expression related to the ICD, and with a method known as consensus clustering, we have detected two different subtypes of BC. The low ICD expression subtype predicted a good prognosis compared to the subtype of high ICD expression. Moreover, four specific genes connected to ICD were utilized to build a predictive risk signature aimed at classifying high- and low-risk BC patients. Our analyses indicate that the ICD gene-based risk signature has good prognostic significance in terms of OS and might therefore be used as an independent predictive marker individual with for BC.

A conceptual framework focusing on ICD mechanisms and ICD- correlated genes was reported by Garg et al. in 2015^33^. In this work, we incorporated additional ICD-related genes through an extensive literature survey and finally identified 41 ICD-correlated genes connected to survival in subjects with BC and other tumors. Upon further analysis, we selected five of the above 41 ICD-related genes, namely *ATG5, CD8A, CD8B, CXCR3*, and *HSP90AA1*, which are closely correlated with the prognosis of BC patients. In fact, the prognostic model consisting of these five genes has the potential to predict the therapeutic efficacy and clinical outcome of breast cancer patients. The high expression of HSP90AA1 and ATG5 in breast cancer patients predicts poor prognosis and poor clinical outcome of tumor patients. In contrast, the amount of CD8 and CXCR3 in breast cancer patients may be correlated with immunotherapy efficacy and predict better clinical outcomes. Therefore, HSP90AA1 and ATG5 in our model may have the potential to be used as biomarkers for early diagnosis and prognostic assessment of breast cancer patients, and by detecting their expression it may be possible to monitor the dynamic progression of breast cancer and assess the effect of immunotherapy in breast cancer patients. Overall, the analysis of this model and genetic testing of tumor patients can predict the quality of survival and therapeutic efficacy of tumor patients in order to provide more references for clinical decision-making.

Tumor therapy may remodel the TME by inducing ICD^34–36^. Mechanistically, ICD is correlated with the exposure and release of DAMPs, which promotes their interaction with certain innate immune cells exhibiting cognate PRRs such as T cells, macrophages, and lymphocytes. This mechanism facilitates the stimulation, maturation, and migration of immune cells towards lymph nodes that are connected to tumors and contain peptides that are specific to antigens produced from cancer. Subsequently, tumor antigens are exposed to T cells, which enhances infiltration of immune cells in the TME to restrict tumor growth. According to this theoretical framework, we conducted a study of gene expression to identify two subtypes of ICD employing a method called consensus clustering.

Subsequently, we experimentally validated the effects of prognosis-related genes on breast cancer progression to judge the accuracy of the prognostic models. Our data reveal that high HSP90AA1 expression supports the BC cells’ growth, migration, and invasion. In past studies, HSP90AA1 expression also favoured chemoresistance in osteosarcoma and distant metastasis in hepatocellular carcinoma by a mechanism that may be connected to PI3K/Akt/mTOR signaling and epithelial mesenchymal transition^37–39^. However, the specific mechanisms by which HSP90AA1 promotes its proliferation in breast cancer and how it affects immunogenic cell death through immune modulation still warrant investigation.

In conclusion, our investigation highlights the connection between subtypes of ICD and the dynamic evolution of the TME in BC and describes a novel ICD-related prognostic model for risk stratification and immunotherapy efficacy prediction. These results may help advance the study of immunotherapeutic options for BC patients (Supplementary Information).

### Supplementary Information

Supplementary Information.

## Data Availability

Publicly available datasets were analyzed in this study. This data can be found here: https://portal.gdc.cancer.gov/ (TCGA-BRCA) and https://www.ncbi.nlm.nih.gov/geo/query/acc.cgi?acc=GSE20685 (GEO).

## Abbreviations

BC: Breast cancer
DAMP: Damage-associated molecular pattern
DEGs: Differentially Expressed Genes
GEO: Gene Expression Omnibus
GO: Gene Ontology
GSEA: Gene Set Enrichment Analysis
HLA: Human leukocyte antigen
HMGB1: High mobility group proteins
ICD: Immunogenic cell death
KEGG: Kyoto Encyclopedia of Genes and Genomes
KM: Kaplan–Meier
NK: Natural killer
OD: Optical density
OS: Overall survival
PRRs: Pathogen recognition receptors
qRT-pcr: Quantitative real-time PCR
SDS-PAGE: SDS polyacrylamide gel electrophoresis
SNPs: Single nucleotide polymorphisms
TIDE: Tumor Immune Dysfunction and Exclusion
TME: Tumor microenvironment
