# Supplementary figures and images for: Immunogenic cell death-related classification reveals prognosis and effectiveness of immunotherapy in breast cancer
Source: Sci Rep. 2024 Jan 23;14:2025. doi: 10.1038/s41598-024-52353-4 (PMC10805874; doi:10.1038/s41598-024-52353-4)

**Figure 9B**  
HSP90AA1

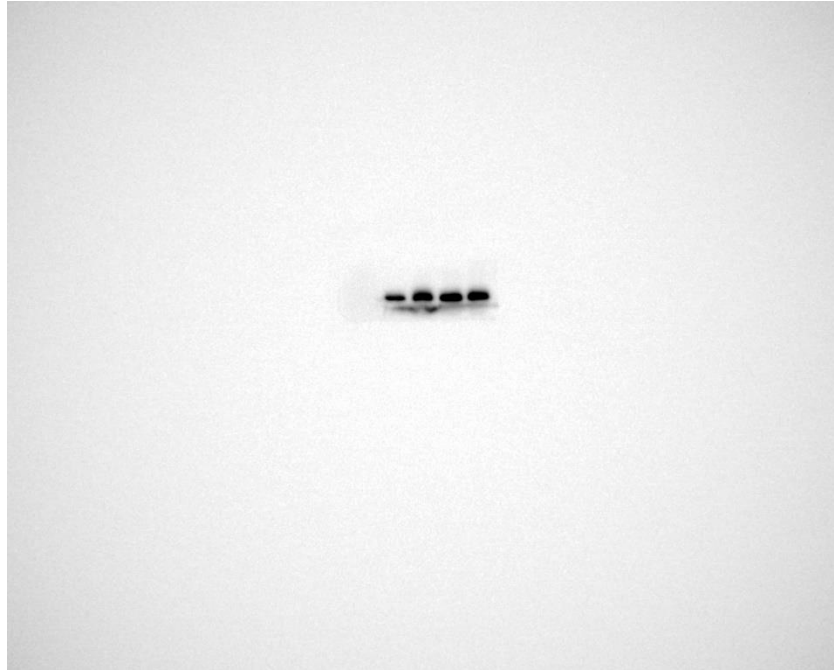

$\beta$ -actin

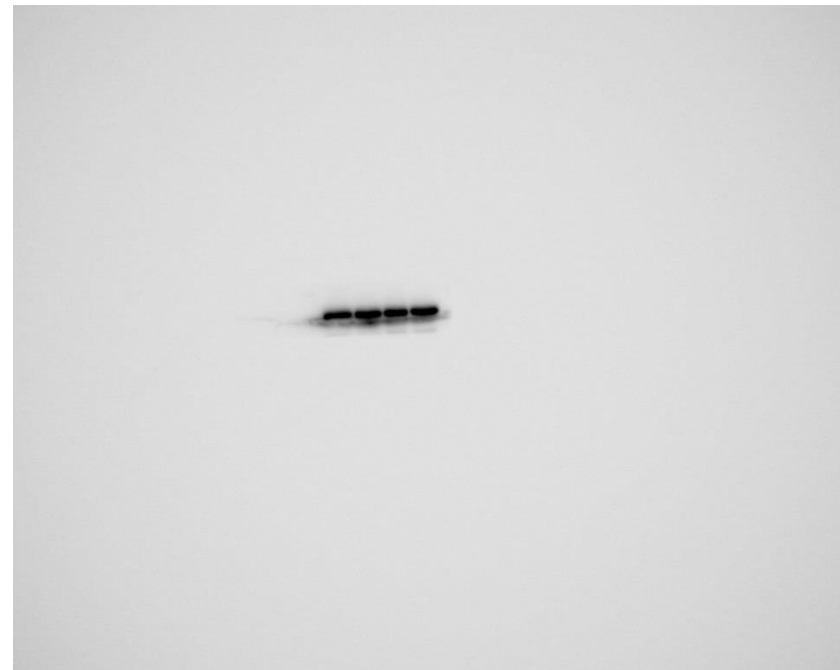

Figure 9D  
HSP90AA1

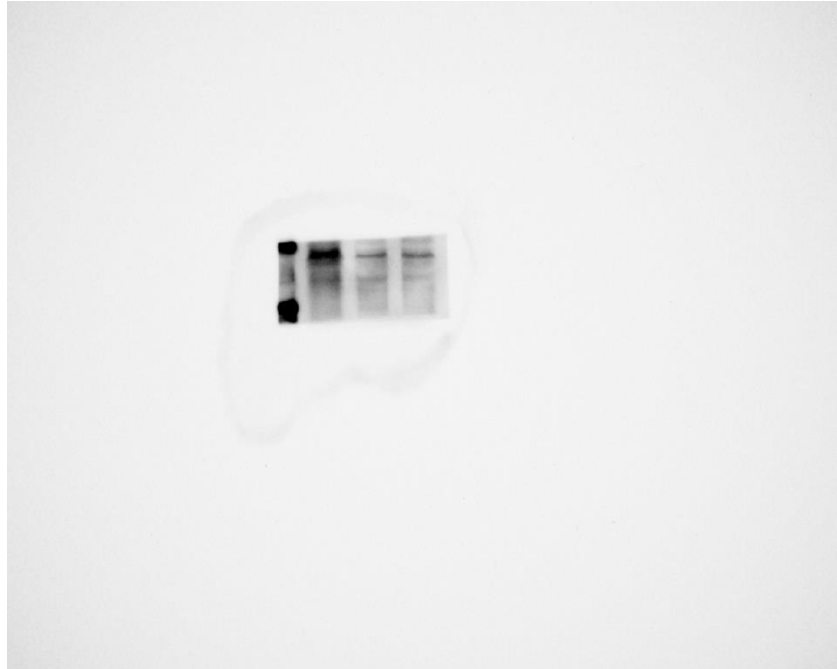

$\beta$ -actin

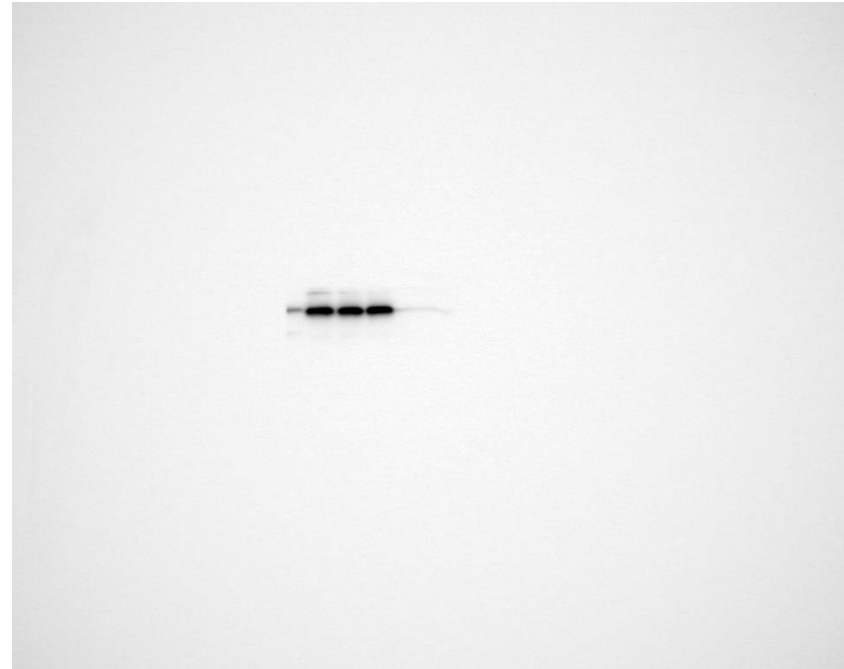

HSP90AA1

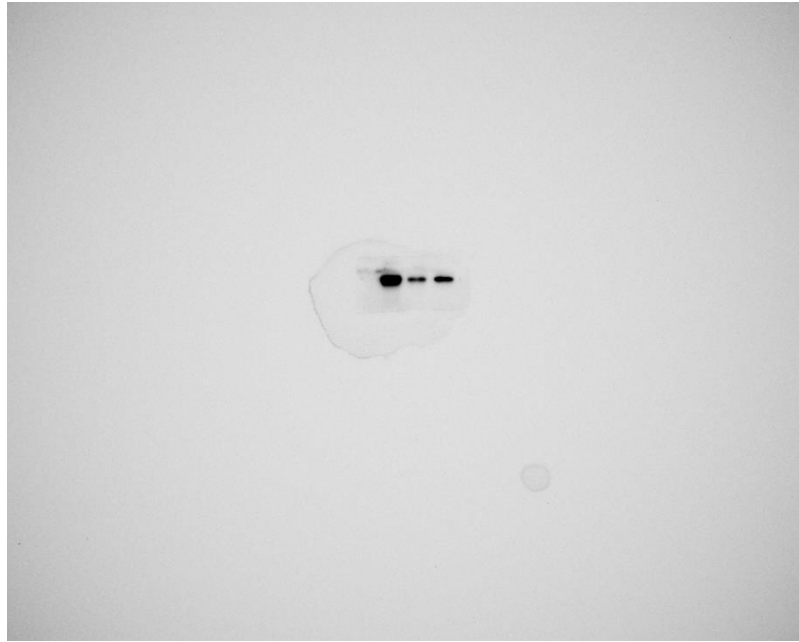

$\beta$ -actin

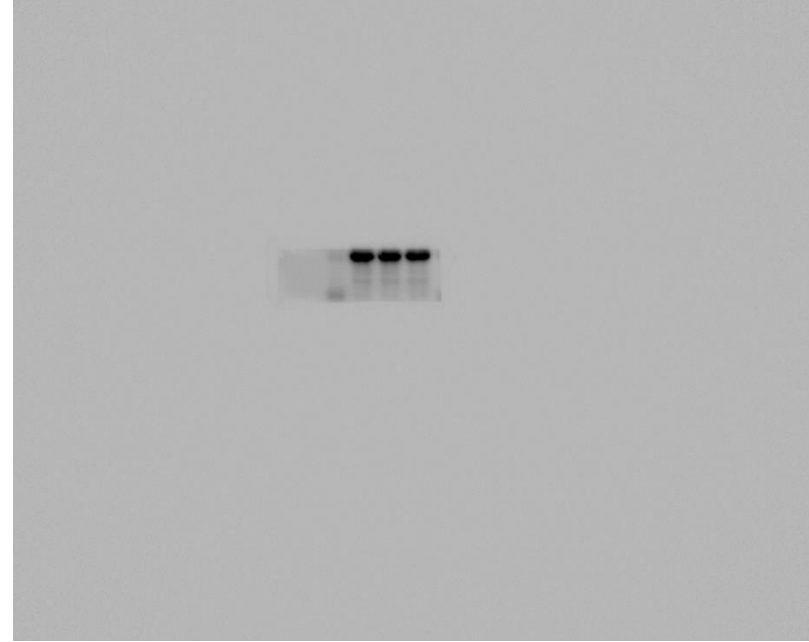

Supplement: Supplementary file 1 — Supplementary Information. [file 41598_2024_52353_MOESM1_ESM.pdf]
